# Supplementary material for: Different Resistance to DON versus HT2 + T2 Producers in Nordic Oat Varieties
Source: Toxins (Basel). 2022 Apr 28;14(5):313. doi: 10.3390/toxins14050313 (PMC9144082; doi:10.3390/toxins14050313)
Supplement: Supplementary file 1 [file toxins-14-00313-s001.zip › toxins-1682935-supplementary.pdf]

Supplementary info

Table S1: Overview of the different field trials in which mycotoxin and/or Fungal DNA content were quantified and included in the statistical analysis.

Field (Year and site number)	DON[1]	HT2+T2[2]	Fungal DNA[3]	FA[3]	FG[3]	FCFG[3]	FL[3]	FP[3]	MM[3]	MN[3]
2011-1	x	NA								
2011-2	x	NA								
2011-3	x	NA								
2011-4	x	NA								
2011-6	x	NA								
2011-7	x	NA								
2011-8	x	NA								
2012-1	x	NA								
2012-4	x	NA								
2012-5	x	x								
2012-7	x	x								
2012-8	x	NA								
2013-4	NA	x	x	x			x			
2013-7	NA	(x)	x	x	x	x	x			
2013-8	NA	(x)	x				x	x		
2014-3	NA	x	x	x			x	x		
2014-4	NA	x	x	x			x	x		x
2014-5	NA	x	x	x	x	x	x	x		x
2015-4	x	NA	x	x	x	x	x	x	x	x
2015-5	NA	x	x	x			x	x	x	x
2015-6	NA	x	x	x			x	x		
2015-7	x	NA	x				x	x		
2015-9	x	NA	x	x	x	x	x	x	x	x
2016-3	x	NA	x	x	x	x	x	x	x	x
2016-7	x	NA	x	x	x	x	x	x	x	
2017-10	x	NA	x	x	x	x			x	x
2018-3	(x)	x	x	x	x	x	x	x		
2019-2	x	x	x	x	x	x	x	x	x	
2019-5	(x)	x	x	x			x	x		
2019-6	x	(x)	x	x	x	x	x	x		
2019-9	NA	x	x	x			x	x		
2019-10	(x)	x	x	x			x		x	
2020-2	(x)	x								
Total number of fields	24	17	20	18	10	10	19	16	8	7

1: X=The content of DON was above 100 µg/kg in grains harvested from variety Belinda. (X) = DON below 100 µg/kg in Belinda. NA=not analysed

2: X= The content of HT2+T2 was above 25 µg/kg in grains harvested from variety Belinda. (X) = HT2+T2 below 25 µg/kg in Belinda. NA=not analysed

3: The DNA content of *Fusarium avenaceum* (FA), *Fusarium culmorum* (FC), *Fusarium graminearum* (FG), *Fusarium langsethiae* (FL), *Fusarium poae* (FP), *Microdochium majus* (MM), and *Microdochium nivale* (MN) in grains from all the fields harvested in years 2013-2019 was analysed by qPCR. Only data from fields in which Belinda had a DNA content of more than 20 pg/µg of the specific fungal species were included in the statistical analysis of possible significant differences in fungal DNA content between varieties (indicated by a separate column for each fungal species).

Table S2: Estimated average levels of mycotoxins (ug/kg) and fungal DNA (pg fungal DNA per µg plant DNA) in oat varieties and breeding lines

Variety or breeding line	DON[1,5]	Group [2]	N[3]	HT2+ T2[1,5]	Group [2]	N[3]	Mycotox years	FA[4]	Group	N	FCFG [4]	Group	N	FG[4]	Grou p	N	FL[4]	Group	N	FP[4]	Group	N	MM [4]	Gr ou p	N	MN[4]	Group	N
Akseli	297	DE	12	148	ABCDE	6	2011-14	197	AB	5							645	ABCD	6	134	AB	4						
Avanti	968	AB	12	230	ABCDE	3	2011-13			<3							449	ABCD	3									
Avetron	176	E	8	126	BCDE	12	2013-20	84	AB	18	34	A	10	33	A	10	419	BCD	19	51	AB	16	118	A	8	6	C	13
Belinda	777	ABC	20	287	AB	14	2011-20	194	AB	18	151	A	10	123	A	10	586	AB	19	76	AB	16	240	A	8	39	A	13
Caddy			<3	220	ABCDE	4	2018-19	154	AB	5							620	ABCD	5	48	AB	4				11	ABC	3
Canary			<3	99	BCDE	4	2019	230	AB	5							477	ABCD	5	77	AB	4				11	ABC	3
Delfin	419	ABCDE	6			<3	2015-17	245	AB	6	90	A	4	89	A	4	456	ABCD	6	86	AB	6	220	A	4	42	AB	6
Dovre	255	BCDE	5	176	ABCDE	6	2012-14	104	AB	5							373	ABCD	6	35	AB	4						
Eidskog			<3	130	BCDE	6	2018-20	147	AB	6	61	A	3	18	A	3	462	ABCD	6	74	AB	5				12	ABC	4
Gimse	434	BCDE	12	187	ABCDE	6	2011-14	104	AB	5							467	ABCD	6	105	AB	4						
GN 07133	1511	A	7			<3	2011			<3																		
GN 07134	759	ABCD	7			<3	2011			<3																		
GN 08009	600	ABCDE	12	104	ABCDE	3	2011-13			<3							490	ABCD	3									
GN 09078	1134	ABCD	5	432	ABC	3	2012-13			<3							750	ABCD	3									
GN 09111			<3	121	ABCDE	4	2013-14	159	AB	5							278	BCD	6	70	AB	4						
GN 11135			<3	157	ABCDE	3	2014	236	AB	3							437	ABCD	3	102	AB	3						
GN 12230	852	ABCD	6			<3	2015-17	105	AB	7	91	A	5	90	A	5	531	ABCD	7	69	AB	7	237	A	6	13	ABC	7
GN 14070			<3	132	ABCDE	5	2019-20	109	AB	6							408	ABCD	6	93	AB	5				14	ABC	4
GN 14182			<3	145	ABCDE	6	2019-20	111	AB	6	97	A	3	106	A	3	316	BCD	6	29	AB	5				14	ABC	4
GN 14189			<3	157	ABCDE	6	2019-20	141	AB	6							452	ABCD	6	73	AB	5				15	ABC	4
GN 14209			<3	180	ABCDE	6	2019-20	261	AB	6	42	A	3	45	A	3	733	ABCD	6	89	AB	5				14	ABC	4
GN 15154			<3	205	ABCDE	5	2019-20	101	AB	6	44	A	3	48	A	3	539	ABCD	6	58	AB	5				15	ABC	4
GN 16061			<3	165	ABCDE	5	2019-20	158	AB	5							609	ABCD	5	128	AB	4				11	ABC	3
GN16165			<3	278	ABCDE	5	2019-20	195	AB	5							673	ABCD	5	67	AB	4				11	ABC	3
GN 16174			<3	112	BCDE	5	2019-20	114	AB	5							297	BCD	5	48	AB	4				34	ABC	3
Gunhild			<3	276	ABCD	6	2018-20	245	AB	6	130	A	3	57	A	3	393	ABCD	6	45	AB	5				12	ABC	4

Haga	437	BCDE	20	176	BCDE	14	2011-20	164	AB	18	75	A	10	57	A	10	464	ABCD	19	115	A	16	233	A	8	13	ABC	13
Hurdal	297	DE	12	128	BCDE	6	2011-14	68	AB	5							517	ABCD	6	90	AB	4						
Hurum	364	CDE	18	81	E	9	2011-18	81	AB	13	95	A	8	89	A	8	281	CD	14	48	AB	12	142	A	6	16	ABC	10
Ivory	963	AB	12			<3	2011-12																					
LW 06W146-02			<3	295	ABCDE	3	2014	123	AB	3							546	ABCD	3	42	AB	3						
Mirella	772	ABCD	12	130	ABCDE	3	2011-13			<3							625	ABCD	3									
Moby			<3	322	ABCD	4	2013-14	388	A	5							749	ABC	6	72	AB	4						
Nike			<3	177	ABCDE	4	2013-14	128	AB	5							336	ABCD	6	61	AB	4						
Odal	367	CDE	20	419	A	14	2011-20	123	AB	18	31	A	10	31	A	10	889	A	19	78	AB	16	186	A	8	10	BC	13
Poseidon			<3	219	ABCDE	4	2013-14	330	AB	5							471	ABCD	6	55	AB	4						
Ridabu			<3	190	ABCDE	6	2018-20	90	AB	6	19	A	3	17	A	3	548	ABCD	6	106	AB	5				21	ABC	4
Ringsaker	422	BCDE	20	158	BCDE	14	2011-20	135	AB	18	28	A	10	23	A	10	453	BCD	19	101	A	16	108	A	8	12	ABC	13
Skarnes	404	BCDE	12	165	ABCDE	6	2011-14	148	AB	5							405	ABCD	6	62	AB	4						
Staur	235	CDE	6	118	ABCDE	3	2015-18	146	AB	8	73	A	6	70	A	6	748	AB	8	106	AB	8	253	A	6	16	ABC	8
Symphony	808	ABCD	12	126	ABCDE	3	2011-13			<3							420	ABCD	3									
Vinger	338	DE	20	102	DE	14	2011-20	134	AB	18	36	A	10	35	A	10	332	BCD	19	46	AB	16	105	A	8	12	ABC	13
Våler	455	ABCDE	13	106	CDE	14	2012-20	135	AB	18	36	A	10	36	A	10	278	D	19	42	AB	16	203	A	8	21	ABC	13
Årnes	442	ABCDE	6	264	ABCD	7	2013-18	68	B	13	46	A	8	43	A	8	483	ABCD	14	26	B	12	84	A	6	18	ABC	10
Range[6], P(variety)[7]:	176-1511	<0.001		81-419	<0.001			68-388	0.001		19-151	0.12		17-123	0.10		278-889	0.001		26-134	0.004		84-253	0.003		6-42	0.002	
N[8]	25			39				36			18			18			41			36			12			25		

1: Estimated average levels of deoxynivalenol (DON) and HT2+T2-toxins (in µg/kg) of a variety, based on Mixed effects model and Tukey analysis in Minitab.
2: Means that do not share a letter are significantly different according to Tukey Method and 95% Confidence.
3: N= number of field sites from which data for a specific variety was included in the analysis.
4: Estimated average levels of fungal DNA (pg fungal DNA per µg plant DNA) in grains harvested from a variety, based on Mixed effects model and Tukey analysis in Minitab. The DNA content of the following fungal species were analysed by quantitative PCR: Fusarium avenaceum (FA), Fusarium culmorum (FC), Fusarium graminearum (FG), Fusarium langsethiae (FL), Fusarium poae (FP), Microdochium nivale (MN) and Microdochium majus (MM).

5: Ln transformed data of mycotoxins or fungal DNA contents were used as input in the statistical analysis (Mixed effects model in Minitab). The values shown in this table are back transformed data of the mean ln output for each variety calculated by Mixed effects model and Tukey pairwise comparison in Minitab.
6: Range min-max value in the dataset
7: P value for variety effects calculated by Mixed effects model in Minitab
8: The number of oat varieties and breeding lines that were included in the statistical analysis

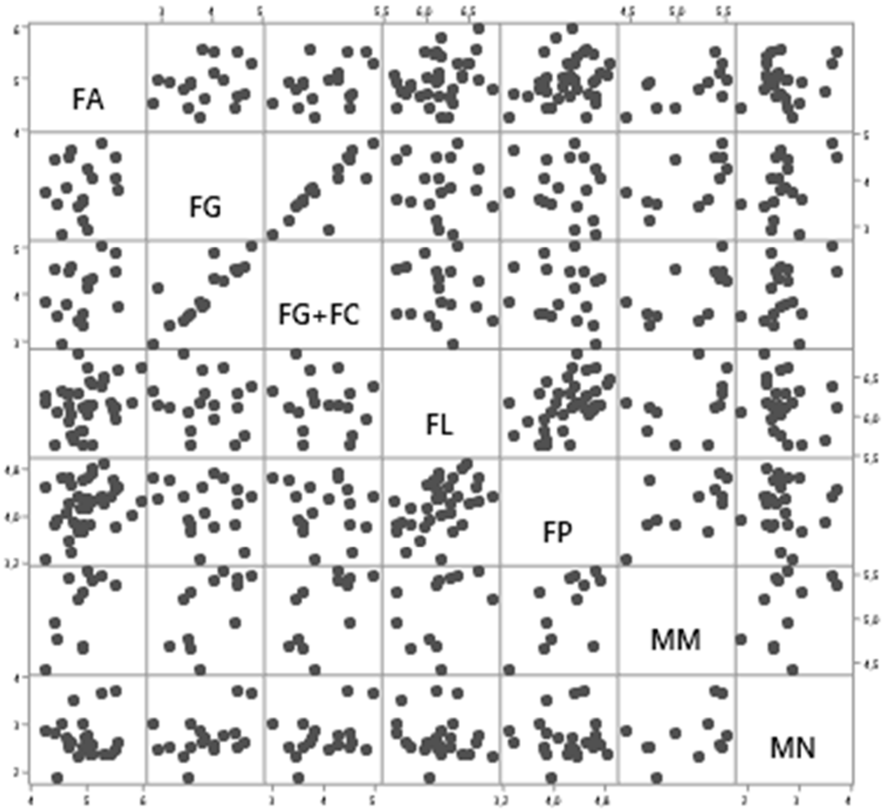

Figure S1: Matrix plot showing the ranking of oat varieties and breeding lines according to average fungal DNA levels in harvested grains (ln pg fungal DNA per µg plant DNA) estimated for: *F. avenaceum* (FA), *F. graminearum + F.culmorum* (FG+FC), *F. langsethiae* (FL), *F. poae* (FP), *M. majus* (MM) and *M. nivale* (MN). Fungal DNA content was analyzed in grains harvested from plants grown in naturally infested field trials in Norway, years 2013-2019. The estimated average mycotoxin or fungal DNA level for each variety included in this analysis is the output from Tukey pairwise comparisons after statistical analysis by mixed effects models in Minitab. The name of the different oat varieties and breeding lines are not indicated in the figure.
